# Correction to “Natural Negative Feedback Loops Confer *Indica*‐*Japonica* Differentiation for Grain Size Homeostasis in Rice”

**DOI:** 10.1002/advs.76296

**Published:** 2026-07-20

X. Li, M. Wu, Z. Qiao, et al. “Natural Negative Feedback Loops Confer Indica‐Japonica Differentiation for Grain Size Homeostasis in Rice.” *Advanced Science* 13, no. 19 (2026): e16180. https://doi.org/10.1002/advs.202516180.

We find that the funding No. (2026AFA004) of the project (Hubei Provincial Natural Science Foundation of China (Outstanding Research Group Project) was missed in the Acknowledgements. The funding information is mentioned below:

Hubei Provincial Natural Science Foundation of China (Outstanding Research Group Project, 2026AFA004).

We apologize for this error.

